# Photovoice-Studie CORONA: Erfahrungen aus dem Leben der Hochrisikogruppe während der Pandemie

**DOI:** 10.1007/s11553-021-00912-2

**Published:** 2021-11-08

**Authors:** Saskia Redlof, Madlen Götz, Marlen Niederberger

**Affiliations:** Pädagogische Hochschule Schwäbisch Gmünd, Forschungsmethoden in der Gesundheitsförderung und Prävention, Oberbettringer Straße 200, 73525 Schwäbisch Gmünd, Deutschland

**Keywords:** COVID-19, Chronische Grunderkrankung, Gesundheitskompetenz, Vulnerable Zielgruppen, Wohlbefinden, COVID-19, Chronic disease, Health literacy, Vulnerable target group, Well-being

## Abstract

**Hintergrund:**

Personen, die der Hockrisikogruppe angehören, sind einem deutlich erhöhten Risiko ausgesetzt bei einer Infektion mit COVID-19 („coronavirus disease 2019“) einen erschwerten Krankheitsverlauf zu entwickeln und daran zu sterben. Um die Verbreitung von COVID-19 in der Bevölkerung einzudämmen, werden situationsangepasste Maßnahmen durchgesetzt, die Änderungen der Alltagsgestaltung bewirken.

**Ziel der Arbeit:**

Die Auseinandersetzung der Hochrisikogruppe mit den Auswirkungen der Coronapandemie, die partizipative Erforschung ihrer Alltagsgestaltung, Sorgen und Gesundheitsressourcen stehen im Zentrum dieser Studie.

**Material und Methoden:**

Zur Analyse der Lebenswelt der Hochrisikogruppe wurde die Photovoice-Methode im Online-Format mit drei Workshops eingesetzt. Sieben Hochrisikopatient:innen mit unterschiedlichen Grunderkrankungen schildern, was Corona für sie im Alltag bedeutet und wofür sie dennoch dankbar sind. Die Rekrutierung erfolgte über persönliche Kontakte des Forschungsteams.

**Ergebnisse:**

Die Co-Forschenden haben neun Geschichten formuliert, anhand derer ihre Dankbarkeit gegenüber des Gesundheitssystems und sozialen Umfelds zum Ausdruck kommt. Die Frage einer gesellschaftlichen Stigmatisierung von Personen mit Grunderkrankungen wird kritisch reflektiert. Die Geschichten zeigen, dass sie gesundheitskompetent handeln und persönliche Gesundheitsressourcen bewusst einsetzen, um negative Folgen auf ihre Gesundheit zu vermeiden und ihr Wohlbefinden zu steigern.

**Diskussion:**

Die Co-Forschenden erweisen sich als gesundheitskompetent und in der Lage, ihr Wohlbefinden positiv zu beeinflussen. Es deutet sich jedoch an, dass der gesellschaftliche Umgang mit vulnerablen Gemeinschaften weiter zu erforschen ist, insbesondere mit Blick auf Diskriminierungsprozesse und einer bedarfsgerechten Gesundheitsversorgung.

## Abstract (en)

**Background:**

People who belong to the high-risk group are exposed to develop a more severe course of the coronavirus disease and have an increased risk of death if they are infected by COVID-19. To limit the spread of COVID-19 among the population, situationally appropriate regulations are enforced that change the way people live their daily lives.

**Objective:**

How does the high-risk group deal with the impact of the coronavirus pandemic, the participatory research of their everyday organization, concerns and own health resources, are the focus of this study.

**Material and methods:**

The photovoice method was used in an online format to analyze the living environment of the high-risk group. In this context three online workshops were conducted. Seven high-risk patients affected by different chronic diseases describe with pictures and stories what COVID-19 means to them and what they are grateful for in this exceptional situation. The recruitment was done by personal contact of the research team with potential participants.

**Results:**

The co-researchers have formulated nine stories expressing their feelings of gratitude towards the healthcare system and their social environment. The question of a social stigmatization of persons with underlying diseases is critically reflected.

The high-risk patients act in a health-competent way, consciously apply personal health resources, such as social contacts or health-promoting daily routines, to avoid negative consequences on their health and to increase their well-being.

**Discussion:**

The co-researchers show a health-competent lifestyle and are able to influence their well-being in a positive way; however, there are indications that the long-term approach to vulnerable communities needs to be discussed, especially concerning discrimination processes and a needs-based health care.

Die Gefahr eines schwerwiegenden Krankheitsverlaufs bei einer Infektion mit COVID-19 („coronavirus disease 2019“) ist für bestimmte Bevölkerungsgruppen deutlich erhöht. Dies führt dazu, dass den Betroffenen zu verstärkter Vorsicht im Alltag geraten wird und deutschlandweit verschiedene Maßnahmen initiiert werden, um gerade diese Gruppen zu schützen. Welche Auswirkungen Corona auf den Alltag der Hochrisikogruppe während der Pandemie hat, welche Ängste sie begleiten und woraus sie dennoch Kraft schöpfen können, wird in diesem Beitrag aus Sicht der Betroffenen diskutiert.

Personen mit bestimmten Grunderkrankungen sind einem deutlich erhöhten Risiko ausgesetzt, bei einer Infektion mit COVID-19 einen erschwerten Krankheitsverlauf zu entwickeln [8]. Zur Hochrisikogruppe zählen nach dem Robert Koch-Institut (RKI) u. a. Personen mit Erkrankungen des Herz-Kreislauf- und Atmungssystems, Diabetes, Krebserkrankungen etc. [20]. Aber auch weitere Faktoren, die den Gesundheitszustand beeinträchtigen, wie Adipositas, Rauchen, ein geschwächtes Immunsystem oder Personen im höheren Lebensalter (50 Jahre und älter) stuft das RKI als deutlich gefährdet ein. Schätzungen zur Folge ist rund ein Drittel der deutschen Bevölkerung ab 15 Jahren der Hochrisikogruppe zuzuordnen [22]. Dies sind häufig Personen mit niedrigem sozioökonomischem Status [27].

Um die Verbreitung von COVID-19 in der Bevölkerung einzudämmen, werden während der Coronapandemie durch die Politik, beratend und unterstützt von der Wissenschaft, kontinuierlich situationsangepasste Regelungen durchgesetzt, wie die Maskenpflicht oder die Schließung von Bildungseinrichtungen [2].

Durch die damit einhergehenden Veränderungen in der Alltagsgestaltung ist auch die Gesundheit derer betroffen, die nicht an COVID-19 erkranken [1]. So zeigt beispielsweise eine bundesweite Studie, dass die Coronakrise stark belastende Auswirkungen auf die psychische Gesundheit von Kindern und Jugendlichen hat [25]. Weitere Studien belegen negative Auswirkungen auf die Gesundheit und die Lebenszufriedenheit berufstätiger Eltern [10], Studierender bzw. Hochschulbeschäftigter sowie auf Erwerbstätige [17].

Die aktuell verfügbare Datenlage deutet an, dass sich bisherige Studien über Hochrisikopatient:innen auf Personen mit spezifischen Krankheitsbildern wie Herz- oder Krebserkrankungen konzentrieren [13, 16]. Wie sich die Pandemie auf das Wohlbefinden von Hochrisikopatient:innen mit unterschiedlichen Grunderkrankungen auswirkt, widmet sich die folgende Studie.

## Das Forschungsprojekt

Partizipative Gesundheitsforschung (PGF) gewinnt in Deutschland an Bedeutung [32]. Assoziierte Ziele sind positive Veränderungen auf die Gesundheit der Vertreter:innen einer Community (Co-Forschende) und die Unterstützung bei der Schaffung gesundheitsfördernder Verhältnissen in ihrer Lebenswelt [7].

Dem Ansatz der PGF folgen diverse Studien im Kontext von Corona, beispielsweise zum Thema Wohlbefinden von Studierenden oder Auswirkungen von Distanzlernen auf Kinder [11, 12]. Derartige Studien belegen auf einer methodischen Ebene, dass PGF auch unter Pandemiebedingungen (beispielsweise als Online-Format) durchführbar ist. Dies zeigt sich insbesondere bei Photovoice-Studien [3, 24]. Bei dieser Art der visuellen Datenerhebungsmethode erstellen Co-Forschende Fotos und ergänzen sie mit Geschichten aus ihrer Lebenswelt [28]. Damit assoziiert ist das Ziel, positive Veränderungen des Gesundheitszustandes der Community zu unterstützen [7].

Im vorliegenden Forschungsprojekt wurde Photovoice zur Analyse der Lebenswelt von Personen der Hochrisikogruppe während der Coronapandemie eingesetzt. Das Forscherteam bestand aus 3 Wissenschaftlerinnen, wobei eine Person selbst als Hochrisikopatientin gilt. Durch sie konnte ein erster niederschwelliger Kontakt zu möglichen Co-Forschenden hergestellt werden. Insbesondere ihre persönlichen Kontakte und der entfernte Bekanntenkreis der angesprochenen Personen wurden genutzt, um über die geplante Studie zu informieren und mögliche Co-Forschende zu gewinnen. Dadurch konnte das Forscherteam ohne einen öffentlichen Aufruf Hochrisikopatient:innen zur Studienteilnahme gewinnen. Die erste Kontaktaufnahme erfolgte über Telefon, E‑Mail sowie durch persönliche Ansprache. Bei der Rekrutierung wurde auf ein möglichst breites Spektrum verschiedenster Grunderkrankungen und Lebensumstände geachtet (Tab. 1), um eine gewisse Heterogenität der Stichprobe zu gewährleisten. Dadurch konnten vielfältige Sichtweisen auf den Untersuchungsgegenstand exploriert werden. Einschlusskriterien waren hierbei, dass sich die Personen als Hochrisikopatient:innen wahrnehmen, ihren Alltag autonom gestalten und in keiner speziellen Pflegeeinrichtung leben.

**Tab. 1:** Charakterisierung der Stichprobe zur Photovoice-Studie CORONA

Name	Alter (Jahre)	Geschlecht	Erkrankung	Lebenssituation
*Magdalena*	25	W	Diabetes Typ III	Alleinstehend
			Neuroendokriner Tumor	Mehrpersonenhaushalt
			Hashimoto	Studentin
*Leo*	34	M	Herzinsuffizienz	Alleinstehend
			Chronische Niereninsuffizienz bei Einzelniere	Mehrpersonenhaushalt
				Technischer Berater
*Regina*	45	W	PSC (primärsklerusierende Cholangitis)	Verheiratet
			Zystenniere	Zwei-Personen-Haushalt
			Nur eine Niere veranlagt	Dozentin (Home-Office)
*Theo*	58	M	Metabolisches Syndrom	Verheiratet
			Folgeerkrankungen nach Schlaganfall, Aneurysma	Zwei-Personen-Haushalt
				Frührentner
*Margit*	62	W	Brustkrebs	Verheiratet
				Zwei-Personen-Haushalt
				Lehrerin
*Franziska*	79	W	Herzschwäche	Verheiratet
			Asthma	Zwei-Personen-Haushalt
			Altersbedingte Krankheiten	Rentnerin
*Josef*	83	M	Lungenödem	Verheiratet
				Zwei-Personen-Haushalt
				Rentner

*M* männlich, *W* weiblich

## Projektdurchführung

**Abb. 1:**
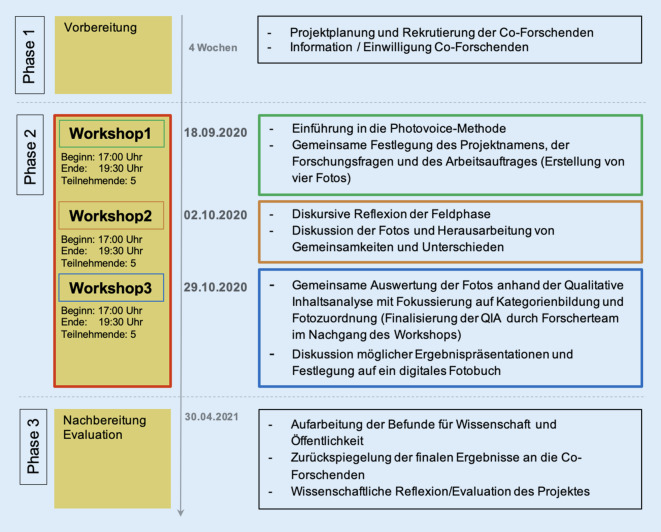
Schematische Darstellung des Projektverlaufs der Photovoice-Studie CORONA

Nach der ersten Kontaktaufnahme mit den Co-Forschenden und dem Einholen einer informierten Zustimmung wurde das Projekt mit drei Online-Workshops durchgeführt, von denen zwei digital aufgezeichnet und transkribiert wurden (Abb. 1). Das Forscherteam hat im Vorfeld der Studie an einer Moderationsschulung teilgenommen. Die Moderation erfolgte durch zwei der Wissenschaftlerinnen auf Basis eines Moderationsleitfadens (s. zusätzliches Online-Material, abrufbar über …) und unter Anwendung verschiedenster Reflexionsmethoden, wie etwa *Blitzlicht* oder *Stimmungsbarometer*. Die Darstellung wichtiger Informationen und relevanter Inhalte erfolgte über eine Powerpoint-Präsentation.

Zudem gab es einen regelmäßigen Austausch via Mail und Telefon, der sowohl durch das Forscherteam als auch durch die Co-Forschenden initiiert wurde. Die Wissenschaftlerinnen traten an die Teilnehmenden heran, um die weiteren Arbeitsschritte zu besprechen und mögliche Unklarheiten oder Bedenken auch bilateral besprechen zu können. Ein wichtiges Anliegen war es auch, die Chance für einen vertrauensvollen Umgang miteinander zu erhöhen. Auch wenn einer der Co-Forschenden an einem Workshop verhindert war, wurde der Kontakt gesucht, um die Ergebnisse zu besprechen, gemeinsam zu reflektieren und ggf. zu ergänzen bzw. zu präzisieren. Am Projektende wurden die Co-Forschenden regelmäßig über die Ergebnisse der wissenschaftlichen Auswertung informiert. Die Co-Forschenden haben sich beim Forscherteam gemeldet, wenn sie an einem Workshop nicht teilnehmen konnten. Jede Kontaktaufnahme wurde schriftlich protokolliert, um die Rückmeldungen mit in die Auswertung einfließen lassen zu können.

Bei den Gesprächen bilateral oder in der Gruppe wurden die Co-Forschenden immer wieder auf ihre Entscheidungsmacht im Projekt hingewiesen und deutlich gemacht, dass die Umsetzung und Ergebnisaufbereitung in ihren Händen liege. Das Forscherteam sei dazu da, zu unterstützen und mögliche Wege aufzuzeigen. Während der gesamten Studie wurde also darauf geachtet, dass die Co-Forschenden, den Partizipationsstufen von Wright [31] folgend, mindestens Mitbestimmung, möglichst aber Entscheidungsmacht erhielten. So haben die Co-Forschenden die Forschungsfragen selbst formuliert:

1. Wofür bin ich dankbar?
2. Was bedeutet Corona für mich?

Die Co-Forschenden nahmen Fotos zu den partizipativ herausgearbeiteten Fragestellungen auf und reflektieren diese innerhalb des strukturierten und moderierten Prozesses während der Workshops. Die Daten wurden über die Fotos, Geschichten, die Protokolle der bilateralen Gespräche und über die gemeinsamen Gruppendiskussionen (festgehalten über Transkriptionen) generiert. Ziel war es auch, die Co-Forschenden zu befähigen, ein tieferes Verständnis über die eigenen Bedarfe und Ressourcen, unter Pandemiebedingungen, zu erlangen [7].

## Auswertung

Die wissenschaftliche Auswertung der Daten erfolgte durch die Wissenschaftlerinnen, unter Anwendung der zusammenfassenden Qualitativen Inhaltsanalyse [15]. Diese erfolgte zunächst unabhängig und wurde im Anschluss innerhalb des Teams der Wissenschaftlerinnen kritisch reflektiert und überarbeitet. Die Kategorien entsprechen den Geschichten der Co-Forschenden, die sie bei den Workshops gemeinsam entwickelt haben. Dazu gehören die von ihnen ausgewählten Fotos und Titel.

Innerhalb gemeinsamer Sitzungen der Wissenschaftlerinnen wurden die Aussagen der Co-Forschenden und mögliche Interpretationen kategorienbezogen diskutiert und reflektiert. Die wissenschaftliche Auswertung verfolgte das Ziel, über die formulierten Geschichten der Co-Forschenden hinaus einen tiefgründigen Einblick zu bekommen und auch Dinge zu erfassen, die nicht direkt von den Co-Forschenden in den Geschichten benannt werden. Den Kodierleitfaden, der zur Qualitativen Inhaltsanalyse entwickelt wurde, ist in Tab. 2 einsehbar.

**Tab. 2:** Kodierleitfaden der qualitativen Inhaltsanalyse

Hauptkategorie	Unterkategorie	Erläuterung	Ankerbeispiel
*HK1:* Eigene Lebenssituation als Hochrisikopatient:in	–	Persönliche Erfahrungen, Meinungen und Verhalten als Hochrisikopatient:in	–
	UK1: Selbstverantwortung/persönlicher Umgang mit Corona (hohe Gesundheitskompetenz)	Der individuelle Umgang mit den Coronamaßnahmen und Hinweise zur Gesundheitskompetenz der Hochrisikopatient:innen	*„Wir können jedoch noch ein wenig Einfluss darauf nehmen, indem wir die Hygieneregeln beachten, außerdem muss man nicht unter die Leute gehen“ (Margit; WS 2)*
	UK2: Eingeschränkter Kontakt zu Familie und Freunden	Hinweise zur Rolle der Freunde und Familie während der Coronazeit, die sowohl positiv wie auch negativ sein können	*„Mittlerweile kann man den Tisch zwar decken, aber es kommt niemand und man sitzt alleine da. Vermutlich weil jeder aus Angst zuhause bleibt“ (Theo; WS 2)*
	UK3: Soziale Isolation und Einsamkeit	Hinweise zu persönlichen Barrieren, Belastungen und negativen Emotionen während der Coronazeit	*„Ich fühle mich isoliert“ (Josef; WS 2)*
	UK4: Notwendigkeit von Achtsamkeit und Selbstfürsorge in einer Ausnahmesituation	Hinweise zu individuellen Ressourcen während der Coronazeit	*„Dankbar bin ich vor allem dafür, dass ich raus gehen kann in unseren Garten“ (Margit; WS 2)*
*HK2:* Positive Aspekte von Corona	–	Durch die Hochrisikogruppe positiv bewertete Sachverhalte und Aktivitäten, die während der Coronazeit festgestellt und entdeckt wurden	–
	UK1: Neue Alltags- und Freizeitgestaltung unter Coronabedingungen	Hinweise zur Veränderung der Freizeitaktivitäten und Verhaltensweisen der Hochrisikopatienten während der Coronazeit	*„Aber jetzt sind wir so drei, vier Kumpels, die dann einfach zusammen spazieren gehen und über alles Mögliche uns austauschen – Früher saßen wir eben immer zusammen bei einem Bier“ (Leo; WS 2)*
	UK2: Bedeutungsgewinn technischer Kommunikationswege	Hinweise zur vermehrten Nutzung von technischen Kommunikationsmitteln und deren Vorteile während der Coronazeit	*„Wir haben meiner Oma ein Handy besorgt und ihr erklärt, wie es funktioniert und das Video-Chatten funktioniert jetzt schon richtig gut“ (Leo; WS 2)*
	UK3: Verständnis für die Schutzmaßnahmen und das Mitwirken der gesamten Bevölkerung	Hinweise zur Wahrnehmung der Hochrisikopatienten von positiven Umständen und Wertschätzung der Gesundheit und Verständnis für die Schutzmaßnahmen innerhalb verschiedener Lebenswelten während der Coronazeit	*„Klar gibt es in der Gesellschaft Menschen, die ein besonderes Risiko haben an Corona zu erkranken und für diese wären die Auswirkungen wahrscheinlich dramatischer als für andere und deswegen brauchen sie einen besonderen Schutz“ (Regina; WS 2)*
*HK3:* Vorurteilsfreie Fremdwahrnehmung von Hochrisikopatient:innen	–	Wahrnehmen von gesellschaftlicher Akzeptanz und Respekt durch die Hochrisikopatient:innen während der Coronazeit	*„Für mich habe ich das so erfahren, dass die Gesellschaft mir Schutzraum gewährt hat, dass ich beispielsweise im Homeoffice arbeiten konnte“ (Regina; WS 2)*
*HK4:* Bewertung und Wahrnehmung des Gesundheitssystems	–	Subjektive Bewertung und Wahrnehmung des deutschen Gesundheitssystems während des Coronazeit durch die Hochrisikopatient:innen	*„Ich denke, dass nicht allen solche Sauerstoffgeräte zur Verfügung stehen und bin einmal mehr dankbar in Deutschland mit einem guten Gesundheitssystem zu leben“ (Regina; WS 2)*

Das Ergebnis der Auswertung wurde anschließend an die Co-Forschenden, mit der expliziten Bitte etwaige Änderungswünsche einzubringen, übermittelt. So kamen letztendlich neun Kategorien zustande, auf Basis derer die neun Geschichten mit dazugehörigen Fotos formuliert wurden (Tab. 3). Drei dieser Geschichten zeigen Infobox 1, 2 und 3.

**Tab. 3:** Übersicht zur Photovoice-Studie CORONA

Die Photovoice-Studie auf einen Blick	
*Themenfeld*	Gesundheit von Hochrisikopatient:innen in der Coronazeit
*Forschungsfragen*	Wofür bin ich dankbar?
	Was bedeutet Corona für mich?
*Laufzeit*	06/2020–03/2021
*Methodisches Vorgehen*	Durchführung von drei Online-Workshops
	Regelmäßiger telefonischer Kontakt zwischen Forschenden und Co-Forschenden
*Ergebnisse*	Formulierung von neun Geschichten, inklusive Fotos mit den Titeln:
	1. Hohe Gesundheitskompetenz
	2. Notwendigkeit von Achtsamkeit und Selbstfürsorge in einer Ausnahmesituation
	3. Vorurteilsfreie Fremdwahrnehmung von Hochrisikopatient:innen
	4. Hohe Wertschätzung des deutschen Gesundheitssystems
	5. Verständnis für die Schutzmaßnahmen und das Mitwirken der gesamten Bevölkerung
	6. Neue Alltags- und Freizeitgestaltung unter Coronabedingungen
	7. Soziale Isolation und Einsamkeit
	8. Bedeutungsgewinn technischer Kommunikationswege
	9. Eingeschränkter Kontakt zu Familie und Freunden

### Infobox 1 Kategorie 1: Hohe Gesundheitskompetenz – *Mit gutem Wissen voran*


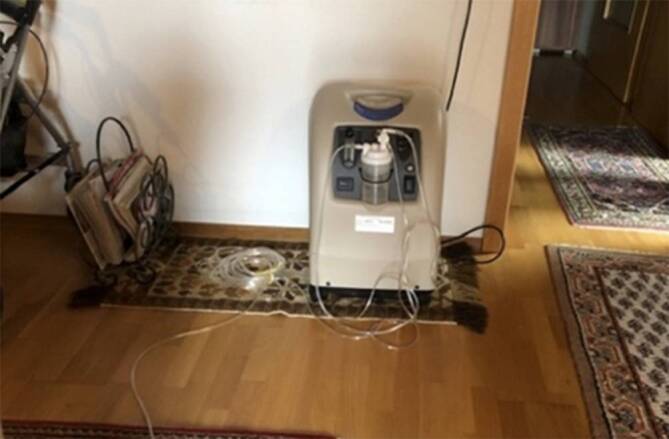


„Mein Sauerstoffgerät hilft mir dabei besser zu atmen und auch zu überleben. Aufgrund meines Lungenödems bekomme ich manchmal sehr schlecht Luft. Das Gerät erinnert mich immer wieder daran, wie gefährlich eine Infektion mit dem Coronavirus für mich wäre. Corona könnte für mich lebensbedrohlich sein. Aber ich bin mir bewusst, dass ich in dieser Situation auch selbst Verantwortung übernehmen kann, um mich vor einer Infektion zu schützen. Ich bleibe zuhause und isoliere mich selbst. Ich kenne die Hygienemaßnahmen, trage draußen eine Maske und finde auch, dass man nicht zwingend unter die Leute gehen muss. Auch Besuch empfange ich nur noch selten und wenn, dann halte ich trotzdem einen großen Abstand ein. Klar würde ich gerne öfter raus gehen, oder meine Familie und Freunde treffen wollen, aber andererseits sollte ich Abstand halten, um mich selbst und andere weniger zu gefährden. Diese Regeln und Auflagen verstehe ich. Ich kann mich selbst vor Corona schützen. Ich muss nicht darauf vertrauen, dass andere die Regeln einhalten. Dadurch habe ich weniger Angst und denke, dass ich diese schwere Zeit überstehen kann.“ – Josef, 83 Jahre, leidet an einer Lungenfunktionsstörung und Diabetes.

### Infobox 2 Kategorie 3: Vorurteilsfreie Fremdwahrnehmung von Hochrisikopatient:innen – *Verständnis füreinander*


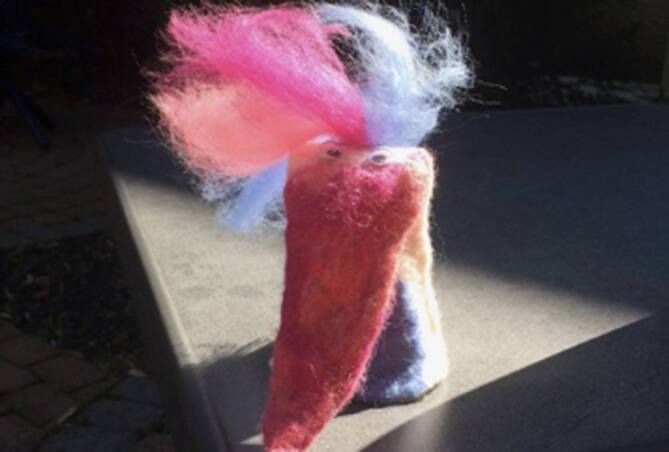


„Diesen Paradiesvogel habe ich selbst gefilzt. Er steht im Licht und leuchtet fröhlich. Corona bedeutet für mich, dass auch Paradiesvögel wie ich wichtig für die Gesellschaft sind und unsere gesundheitlichen Interessen wahrgenommen werden. Denn durch meine chronische Erkrankung gelte ich als Hochrisikopatientin und bin ungewollte ins Rampenlicht gerückt. Aber das ist gut so. Für mich als chronisch Kranke ist in dieser Zeit das Leben sogar leichter geworden. Ich muss mich nicht mehr umständlich erklären, dass ich mehr Rückzug brauche. Ich darf nun im Homeoffice arbeiten. Ich werde geschützt. Ich kann offen zu meiner Krankheit stehen, ohne dafür negativ stigmatisiert zu werden. Ich hoffe, dass dies auch nach Corona so bleibt.“ – Regina, 45 Jahre, leidet an einer primärsklerosierenden Cholangitis und einer Zystenniere.

### Infobox 3 Kategorie 5: Verständnis für die Schutzmaßnahmen und das Mitwirken der gesamten Bevölkerung – *Eine Hand schützt die andere*


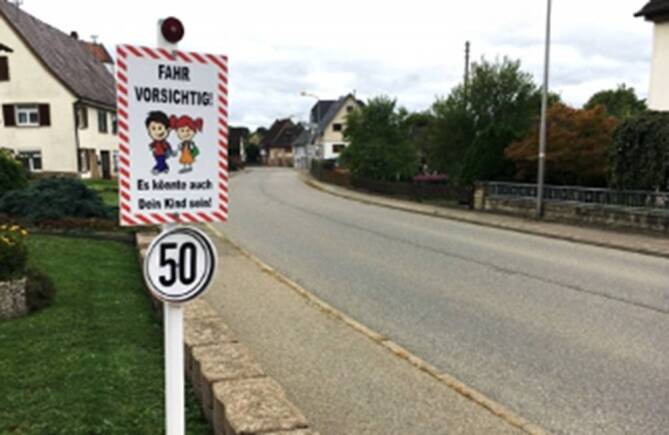


„Das Problem ist, dass jeder ein potenzieller Überträger des Coronavirus sein kann, auch wenn die Person das selbst nicht weiß. Das verunsichert mich und zeigt mir, dass wir gegen das Virus nur gemeinsam ankommen können. Ich bin positiv überrascht darüber, was alles unternommen wurde, um Corona einzudämmen und gerade auch Hochrisikopatient:innen wie mich zu schützen. Jeder kennt vermutlich wenigstens eine Person, bei der man Angst hat, dass sie schwer an Corona erkranken kann. Aber noch mehr erstaunt mich, dass es Menschen gibt, die sich aufopfern und ohne an die eigenen Folgen zu denken, sich um Hochrisikopatient:inenn wie mich kümmern. Ich denk dabei an Pflegekräfte, vor allem an meine Enkelin. Sie ist Krankenschwester und kümmert sich um die Schwachen und Kranken. Ich hoffe, dass auch weiterhin mehr auf die Gesundheit und gegenseitig Aufeinander geachtet wird.“ – Franziska, 79 Jahre, leidet an Asthma und einer Herzschwäche.

## Ergebnisse zu Forschungsfrage 1 „Wofür bin ich dankbar?“

Quer über die Geschichten zeigt sich die Dankbarkeit auf fünf Ebenen:

- Die Co-Forschenden sind über geltende Coronaschutzmaßnahmen informiert, verstehen deren Relevanz bzw. Umsetzung und handeln danach. Sie haben sich im Laufe ihrer Erkrankung die Fähigkeiten angeeignet, sich gesundheitskompetent zu verhalten und persönliche Gesundheitsressourcen zur Förderung ihres Wohlbefindens zu nutzen. Sie sind dankbar, dass sie auf außergewöhnliche Lebensbedingungen flexibel, resilient und achtsam reagieren können. Dies schaffen sie dank individueller Bewältigungsstrategien, wie einer stets reflektierten selbstfürsorgenden Alltagsgestaltung und dem Ausprobieren neuer Hobbys unter Einhaltung der Coronaregelungen.
- Die Co-Forschenden sind dankbar für ihr soziales Umfeld und sind in der Lage, eine gewisse Stabilität ihrer sozialen Kontakte während der Pandemie aufrechtzuhalten. Um mit ihren Großeltern in Kontakt zu bleiben, erläutern sie ihnen die Nutzung digitaler Tools. Dennoch räumen die Co-Forschenden ein, dass ihnen die regelmäßigen und ungezwungenen persönlichen Treffen sehr fehlen.
- Die Co-Forschenden sind dankbar für die Berücksichtigung ihrer gesundheitlichen Belange in der Coronapandemie durch die Gesellschaft. In der Zeit vor Corona machten sie Erfahrungen mit Diskriminierung bzw. Stigmatisierung. In der Ausnahmesituation haben sie nun den Eindruck, dass ihre Gesundheit für Politik und Bevölkerung ein schützenswertes Gut darstellt. Sie kritisieren und verurteilen zwar das Verhalten der „Coronaleugner:innen“, nehmen diese aber als Ausnahme wahr.
- Die Co-Forschenden sind dankbar für die Schutzmaßnahmen, die die Politik auf den Weg gebracht hat, um insbesondere Hochrisikopatient:innen vor einer COVID-19-Infektion zu bewahren. Sie sind überrascht wie weitreichend diese sind und dass selbst, die bis dato unantastbare Schulpflicht, außer Kraft gesetzt wurde. Die Co-Forschenden berichten sogar von Erleichterungen in ihrem Alltag, die mit den Maßnahmen einhergehen. Durch die Möglichkeit im Homeoffice zu arbeiten, brauchen sie sich nicht vor Kolleg:innen zu rechtfertigen, wenn sie eine Pause benötigen. Bei Arztbesuchen sind die Wartezeiten kürzer und aufgrund der Abstandsregelungen haben sie keine Angst mehr, sich bei Patient:innen mit einer anderen Erkrankung anzustecken.
- Die Co-Forschenden sind auch in der Coronazeit in die medizinische Versorgungsstrukturen eingebettet, d. h. sie nehmen regelmäßige Untersuchungen bzw. Therapien wahr. Sie sind dankbar für die stabilen Versorgungsstrukturen und die Menschen, die sich im Gesundheitsbereich engagieren, unabhängig vom eigenen Risiko einer COVID-19-Erkrankung. Sie haben insgesamt den Eindruck, dass ihre Gesundheitsversorgung bedarfsgerechter erfolgt als vor der Pandemie.

Die Dankbarkeit der Co-Forschenden zeigt sich auf individueller, sozialer und struktureller Ebene. Die Co-Forschenden fühlen sich von Gesellschaft und Politik wahrgenommen, wertgeschätzt und beschützt. Sie sind in der Lage, ihren Coronaalltag achtsam und gesundheitsfördernd zu gestalten.

## Ergebnisse zu Forschungsfrage 2 „Was bedeutet Corona für mich?“

Die Co-Forschenden skizzieren verschiedene Aspekte, die sich durch die Coronapandemie für sie verändert haben.

- Auf der einen Seite fühlen sich die Co-Forschenden ausgeglichener, weil sie sich mehr an der frischen Luft bewegen und weniger beruflichen Stress spüren. Gleichzeitig beschreiben sie Gefühle der Einsamkeit. Der eingeschränkte soziale Kontakt und die Ungewissheit bzw. Machtlosigkeit darüber, wie lange die Coronapandemie anhält, wird als belastend beschrieben.
- Die Co-Forschenden führen die Coronaschutzmaßnahmen auf die Berücksichtigung der Belange von Hochrisikopatient:innen zurück und empfinden dies als etwas Neues, was sie in dieser Form nicht erwartet hätten. Eine Reflexion, inwieweit dies Bestand hat und ob auch andere Gründe bei der Entwicklung von Schutzmaßnahmen eine Rolle spielen, findet nicht statt.
- Corona bedeutet für die Co-Forschenden die Notwendigkeit eines stets reflektierten und bewussten Gesundheitsverhaltens. Das Risiko einer Infektion mit COVID-19 nehmen sie als permanente Bedrohung für ihr Leben wahr, vor der sie sich aber auch selbst schützen können.
- Der Alltag der Co-Forschenden hat sich in der Coronazeit verändert. Sie probieren neue Hobbys aus, halten sich mehr in der Natur auf, nehmen sich Zeit für sich und beschreiben sich als achtsam. Sie haben sich mit den veränderten Bedingungen arrangiert und selbstbestimmt neue Alltagsroutinen etabliert. Gleichzeitig betonen sie den Wunsch wieder mehr zu unternehmen, beispielsweise andere Länder zu bereisen.

Die Co-Forschenden bleiben in ihren Darstellungen trotz des Bewusstseins des hohen persönlichen Gesundheitsrisikos positiv. Negative Gefühle formulieren sie zwar, aber immer im Verhältnis zum Risiko eines schwerwiegenden Verlaufs einer möglichen COVID-19-Erkrankung.

## Ergebnisse der Evaluation

Evaluiert wurde das Projekt anhand eines schriftlichen Fragebogens mit offenen und geschlossenen Items (zu Beginn und am Ende des Projekts). Zudem erfolgte ein direktes Nachfragen innerhalb der Workshops sowie bei den bilateralen Gesprächen. Hierbei wurden eine Meinungsabfrage und ein Stimmungsbild unter Zuhilfenahme der Reflexionsmethoden erhoben und im Anschluss inhaltsanalytisch zusammengefasst. Die Auswertung der Projektevaluation durch die Co-Forschenden zeigt eine positive Einschätzung, auch im Hinblick auf den Grad der Beteiligung. Auf die offene Frage, was sie aus dem Forschungsprojekt mitnehmen, schreiben sie u. a.:

- „Ich habe gemerkt, dass es nicht nur mir allein so geht […]. Daraus kann ich auch für mich Kraft schöpfen.“ – Magdalena
- „Am wichtigsten fand ich die Erfahrung, wie schwierig manche aus unserem Projekt die jetzige Situation erleben und sich nicht darüber beschweren.“ – Margit
- „Wenn ich mich an die Regeln halte, könnte man die Krise halbwegs gut überstehen.“ – Theo

## Diskussion

Die Coronapandemie erweist sich für die Co-Forschenden, wie auch für andere Bevölkerungsgruppen, als eine psychische und soziale Herausforderung [1, 18]. Doch dank individueller Gesundheitsressourcen mildern sie negative Auswirkungen auf ihre Gesundheit ab und steigern bewusst ihr Wohlbefinden [30]. Nach der Terminologie des Belastungs-Beanspruchungs-Modells [21] erlauben ihnen die individuellen Ressourcen innerhalb der geltenden Rahmenbedingungen ein Gefühl der Situationskontrolle, was ihre psychische Beanspruchung zum Zeitpunkt der Studie, also zwischen dem ersten und zweiten Lockdown, relativ gering hält. Auch frühere Studien zeigen, dass Personen mit chronischer Erkrankung eine positive Lebenseinstellung formulieren [6] und dies, obwohl ihre gesundheitsbezogene Lebensqualität gegenüber Personen ohne chronische Erkrankung als eingeschränkt gilt [23, 29]. Die Gesundheitskompetenz chronisch kranker Personen erweist sich als eine zentrale Gesundheitsressource in der Coronapandemie. Ein aktueller Review zeigt, dass zu diesem Thema bisher wenige empirische Daten vorliegen und weiterer Forschungsbedarf besteht [5].

Dennoch formulieren die Co-Forschenden auch Gefühle der permanenten Bedrohung und Machtlosigkeit. Das Risiko einer Ansteckung schwebt als „Damoklesschwert“ über ihnen und wird als bedrückend wahrgenommen [19]. Ambivalent sind die Darstellungen der Co-Forschenden beim Punkt Einsamkeit. Einerseits schaffen es die Co-Forschenden, den sozialen Kontakt zu einzelnen Personen aufrechtzuerhalten, andererseits fehlen ihnen persönliche Treffen. Vergleichbare Befunde zeigen auch andere Studien über die Folgen der sozialen Einschränkungen in der Coronapandemie [14].

Neben den Befunden zum aktuellen Wohlbefinden wird deutlich, dass die Hochrisikopatient:innen ein gesellschaftliches Umdenken hinsichtlich vulnerabler Gemeinschaften wahrnehmen [9]. In einer Zukunftsstudie [4] zu den Folgen der Coronapandemie betonen Expert:innen aus Wissenschaft, Wirtschaft und Sozialverbänden die Notwendigkeit einer zukünftig verschärften Diskussion über „lebenswertes Leben“ sowie Gesundheitsleistungen für vulnerable Gruppen.

Auf einer methodischen Ebene verdeutlichen die Erfahrungen, dass Photovoice auch als Online-Format durchführbar ist [3]. Jedoch fokussiert sich der Austausch der Co-Forschenden während der Workshop-Zeit auf das Forschungsthema und damit sinkt die Chance für den Aufbau dauerhafter sozialer Kontakte. Im Hinblick auf eines der zentralen Ziele der PGF, nämlich der Stärkung vulnerabler Communities [26], kann dies kritisch gesehen werden.

## Limitationen

Die Ergebnisse der Photovoice-Studien stellen eine Momentaufnahme aus dem ersten Jahr der Coronapandemie vor der Verfügbarkeit eines Impfstoffs dar. Ob die eher positiven Befunde über die gesamte Dauer bleiben, ist unklar. Trotz des niederschwelligen Zugangs, dem vertrauensvollen Verhältnis und der Offenheit der Co-Forschenden untereinander, kann nicht ausgeschlossen werden, dass aufgrund von sozialer Erwünschtheit, Angst oder Scham bewusst Aspekte zurückgehalten wurden. Inwieweit die Ergebnisse auf Hochrisikopatient:innen mit einer eher geringen Gesundheitskompetenz zutreffen, wäre weiter zu untersuchen.

## Fazit für die Praxis

- Menschen mit chronischen Erkrankungen unterliegen im Alltag dem Risiko von Stigmatisierung und Diskriminierung. In der Coronapandemie haben die befragten Hochrisikopatient:innen ein höheres gesellschaftliches Interesse und ein gewisses Verständnis für ihre gesundheitlichen Belange erfahren.
- Möglicherweise hat Corona die Bevölkerung für die gesundheitlichen Belange vulnerlabler Gruppen sensibilisiert. Dies kann sich zukünftig auf die Umsetzung und Akzeptanz gesundheitsförderlicher bzw. präventiver Interventionen auf Verhaltens- und Verhältnisebene als eine wichtige Grundlage erweisen.
- Partizipative Forschungsansätze erweisen sich auch in Ausnahmesituationen als geeignet, um die Co-Forschenden zur Selbstreflexion des eigenen Verhaltens anzuregen, die Gesellschaft für die Bedarfe unterschiedlicher Communities zu sensibilisieren und dadurch zum Abbau gesundheitlicher Ungleichheiten beizutragen.

## Anhang

**Tab. 4:** Ablauf – Workshop 1

Zeit	Was?	Notizen
*10* *min*	Check-in und Begrüßung, technische Einführung in den Videokonferenzdienst Zoom, Datenschutzhinweise	Kamera an? (freiwillig)
		Ansprache (Akronym)? Du/Sie?
		Einverständniserklärung ausgefüllt und abgegeben?
		Einführung Zoom: Chat/Hand heben/Ton/Kamera
*5* *min*	Vorstellung Forschungsteam	–
*20* *min*	Vorstellungsrunde der Co-Forschenden	Kurze Kennenlernrunde
		Kamera kann freiwillig angemacht werden
		Orientierungsfragen können beantwortet werden (freiwillig) – Ich bin … – Aktuelle Lebenssituation – Aktuelle Beschäftigung – Hobbies – Was bedeutet das Photovoice-Projekt für mich?
*8* *min*	Einführung in die Sicht der Gesundheitsförderung	Definition Gesundheit
		Aspekte der Salutogenese erläutern
		Regenbogenmodell vorstellen
*10* *min*	Was ist Photovoice?	Infos zur Methode und dem Forschungsprozess
		Kurze Darstellung Projekt: „Hauptsache Mensch“
*10* *min*	Überblick über den Ablauf des Forschungsprojekts	Einen Überblick über das Projekt bieten
		Einzelne Schritte durchgehen und erläutern (WS 1, Feldphase, WS 2, Auswertung, WS 3, Präsentation der Ergebnisse)
*10* *min*	Pause mit anschließender Atemübung	Entspannende Musik wird im Hintergrund abgespielt
*25* *min*	Gemeinsame Formulierung der Forschungsfragen und des Projektnamens	Beispielformulierungen als Anstoß verwenden
		Was eint die Gruppe?
		Logo entwerfen? (Brainstorming/Ideen sammeln)
*10* *min*	Arbeitsauftrag vorstellen (Feldphase)	Aufgabe erläutern (Feldphase)
		Leitfragen erläutern und ggf. ein Beispiel geben
		Auf Arbeitsblatt hinweisen, welches im Anschluss an die Co-Forschenden versendet wird
*20* *min*	Erläuterung ethischer/datenschutzrechtlicher und technischer Aspekte des Fotografierens, kreativer Umgang mit dem Medium	Auf Fotofreigabeformular hinweisen
		Technische und kreative Unterweisung durch einen Co-Forschenden
*10* *min*	Zusammenfassung (WS 1)	Rückblick auf den Workshop: – Was haben wir gelernt? – Was haben wir gemeinsam erarbeitet?
*5* *min*	Ausblick und Verabschiedung	Auf den nächsten Termin ausdrücklich hinweisen
		Nochmal eine Möglichkeit für Fragen bieten

**Tab. 5:** Ablauf – Workshop 2

Zeit	Was?	Notizen
*10* *min*	Check-in und Begrüßung	Kamera an? (freiwillig)
		Ansprache (Akronym)?
		Steckbrief schon ausgefüllt und abgegeben?
		Aufzeichnung – Start (Hinweis an die Co-Forschenden)
*3* *min*	Vorstellung der Agenda für WS 2	–
*20* *min*	Gemeinsame Reflexion der Feldphase	Wie verlief das Fotografieren?
		Wie erging es euch bei der Aufgabe? (einfach/schwer/überraschend)
		Gab es Erkenntnisse?
*5* *min*	Überblick über den Ablauf des Forschungsprojekts	Einen Überblick über den weiteren Verlauf des Projekts bieten (ab WS 2): – Was haben wir schon geschafft? – Was liegt noch vor uns?
*30* *min*	Vorstellung der Fotos (Teil 1)	Vorstellung der Fotos innerhalb der Gruppe
		Die Co-Forschenden können uneingeschränkt reden
*10* *min*	Pause mit anschließender Atemübung	Entspannende Musik wird im Hintergrund abgespielt
*20* *min*	Vorstellung der Fotos (Teil 2)	Siehe oben
*20* *min*	Gruppendiskussion	Gemeinsamkeiten der Gruppe finden
		Was eint die Community?
		Jeder kann sich an der Diskussion beteiligen – keine Vorgaben!!
*10* *min*	Reflexion zum Austausch	Fragen: – Wie erging es euch während eurer Fotopräsentation/der gemeinsamen Diskussion? – Seid ihr zufrieden mit eurem Ergebnis? – Was könnt ihr mitnehmen? – Gibt es Wünsche oder Anregungen?
*15* *min*	Gemeinsamer Austausch über die Art der Ergebnispräsentation	Fragen: – Wie sollen die Ergebnisse ausgearbeitet werden? (Ideen/Anregungen/Wünsche)
		Ideen sammeln
*8* *min*	Zusammenfassung (WS 2)	Rückblick auf den Workshop: – Was haben wir gelernt? – Was haben wir gemeinsam erarbeitet?
*7* *min*	Ausblick und Verabschiedung	Gemeinsame Terminvereinbarung (WS 3)
		Nochmal eine Möglichkeit für Fragen bieten

**Tab. 6:** Ablauf – Workshop 3

Zeit	Was?	Notizen
*10* *min*	Check-in und Begrüßung	Kamera an? (freiwillig)
		Ansprache (Akronym)?
		Aufzeichnung – Start (Hinweis an die Co-Forschenden)
*3* *min*	Vorstellung der Agenda für WS 3	–
*5* *min*	Überblick über den Ablauf des Forschungsprojekts	Einen Überblick über den weiteren Verlauf des Projekts bieten (ab WS 2): – Was haben wir schon geschafft? – Was liegt noch vor uns?
*15* *min*	Vorstellung der Analyse und Auswertung der Gruppendiskussion	Alle Fotos nochmal zeigen
		Leitfragen stellen: – Was macht euch als Community aus? – Was sind Besonderheiten? – Was unterscheidet euch eventuell von „Gesunden“?
*30* *min*	Gruppendiskussion Co-Forschende diskutieren und verifizieren die gebildeten Kategorien (Auswertung) – Teil 1	Leitfragen: – Wie soll die jeweilige Kategorie heißen? (Titel der Geschichten) – Fehlt etwas? – Alles korrekt?
		Co-Forschende können frei ihre Meinung äußern und sind am Entscheidungsprozess beteiligt
*5* *min*	Pause	–
*30* *min* Co-Forschende	Gruppendiskussion, Co-Forschende diskutieren und verifizieren die gebildeten Kategorien (Auswertung) – Teil 2	Siehe oben
*15* *min*	Art der Ergebnispräsentation wiederholt mit der Gruppe besprechen	Ideensammlung nochmals vorstellen
		Abstimmung über die finale Art der Ergebnispräsentation (Co-Forschende)
*10* *min*	Logo Abstimmung	Verschiedene Entwürfe werden dargeboten – Abstimmung durch Co-Forschenden
*15* *min*	Stimmungsbild und Abschlussfragen	Offene Fragen klären
		Stimmungsbild einholen (Stimmungsbarometer)
*7* *min*	Abschließende Anmerkungen und Verabschiedung	DANKE für EURE Mitarbeit!
